# BAG3 genetic variant is associated with heart failure with preserved ejection fraction phenotypes

**DOI:** 10.1371/journal.pone.0361206

**Published:** 2026-11-03

**Authors:** Citrawati Dyah Kencono Wungu, Hendri Susilo, Jesselyn Wijaya, Cleodylon R. Susanto, Kevin Kevin, David Nugraha, Delvac Oceandy

**Affiliations:** 1 Department of Physiology and Medical Biochemistry, Faculty of Medicine, Universitas Airlangga, Surabaya, Indonesia; 2 Institute of Tropical Disease, Universitas Airlangga, Surabaya, Indonesia; 3 Department of Cardiology and Vascular Medicine, Faculty of Medicine, Universitas Airlangga, Surabaya, Indonesia; 4 Cardiovascular and Cardiometabolic Science Research Group, Faculty of Medicine, Universitas Airlangga, Surabaya, Indonesia; 5 Department of Cardiology and Vascular Medicine, Universitas Airlangga Hospital, Surabaya, Indonesia; 6 Medical Program, Faculty of Medicine, Universitas Airlangga, Surabaya, Indonesia; 7 Division of Cardiovascular Sciences, Faculty of Biology Medicine and Health, University of Manchester, Manchester, United Kingdom; Shaheed Rajaei Cardiovascular Medical and Research Center: Rajaie Cardiovascular Medical and Research Center, IRAN, ISLAMIC REPUBLIC OF

## Abstract

Genetic variations are involved in determining heart failure (HF) phenotypes. While variants within the *BAG3* gene have been associated with HF in European populations, the impact of specific single nucleotide polymorphisms (SNPs) within the *BAG3* gene in determining phenotypes of HF with preserved ejection fraction (HFpEF) in Southeast Asian populations is unknown. Therefore, in this study, we identified BAG3 SNPs and investigated their biological and clinical relevance in determining HFpEF phenotypes among an Indonesian HFpEF cohort. We conducted a study involving 36 patients with HFpEF and 30 healthy individuals. We detected the presence of rs196326 and rs2240777 SNPs in our cohort. However, the rs2234962 polymorphism, which has been associated with HF and cardiomyopathy in Western populations, was absent in our Indonesian cohort. We found that the rs196326 SNP was significantly associated with relative wall thickness (RWT), with patients carrying the CG genotype displayed significantly lower RWT. Additionally, the rs196326 CG/GG genotypes were correlated with higher serum N-terminal pro B-type natriuretic peptide (NT-proBNP) and lower Caspase-3 levels. Our study showed that the *BAG3* rs196326 polymorphism was significantly associated with HFpEF phenotypes in the Indonesian population. This highlights the importance of identifying population-specific as genetic markers of disease severity.

## Introduction

Heart failure with preserved ejection fraction (HFpEF) is a complex clinical syndrome characterized by heart failure (HF) symptoms but with normal left ventricular ejection fraction (EF > 50%) [1,2]. The global prevalence of HFpEF is increasing, and currently around 50% of patients with HF are classified as HFpEF [3]. Notably, HFpEF is commonly associated with non-cardiac factors, such as diabetes mellitus (DM), obesity, and hypertension [1,3,4]. Effective treatment options for HFpEF are limited, with several studies reporting that medications that are beneficial for heart failure with reduced ejection fraction (HFrEF) do not produce significant improvements in patients with HFpEF [5].

Genetic factors are evidently involved in the pathophysiology of heart diseases including HF. Susceptibility to HF often involves genetic factors, which may be traced through family origin [6]. A recent genome wide association study (GWAS) on hundreds of thousands of participants identified 13 genetic loci that are associated with HF [7]. The *Bcl-2-associated athanogene 3* (*BAG3*) gene is one of the genes that were found to be associated with HF, particularly HFrEF [7]. The human *BAG3* gene*,* which is located on chromosome 10q26.11, encodes a 575-amino-acid co-chaperone protein. It is predominantly expressed in skeletal and cardiac muscles and is essential in the regulation of protein folding, autophagy, and apoptosis, which are essential processes in maintaining cellular homeostasis [8].

One of the most important single nucleotide polymorphisms (SNPs) within the human *BAG3* gene is the rs2234962 polymorphism, which is associated with dilated cardiomyopathy [9,10]. It is also strongly correlated with the occurrence of HF in one of the largest cohorts of the HF GWAS [7]. This variant is located within an exonic region and causes substitution of cysteine with arginine at position 151. Mechanistic analyses suggest that the *BAG3*^*C151R*^ variant affects preferential binding to interacting proteins, leading to alteration in its protective effects against stress [11]. Most of the studies on the *BAG3* rs2234962 genetic polymorphism have been conducted in Caucasian populations, where this variant is relatively common. Data from the 1000 Genomes Project and gnomAD [12,13] indicate that the polymorphic variant of rs2234962 is quite common in European and South Asian populations (~20%), less common in American (~10%) and African populations (~1–5%), and extremely rare in East Asians (<1%).

In this study, we analyzed genetic variants of human *BAG3* gene in HFpEF patients of Indonesian ethnic origin. While we did not detect the occurrence of the rs2234962 SNP in this cohort, we found other variants, rs196326 and rs2240777, which were located in the vicinity of rs2234962. Interestingly, further analyses revealed that rs196326 was associated with the level of *BAG3* mRNA expression and some HFpEF clinical parameters, indicating a possible clinical relevance and functional significance of this SNP in altering key cellular functions. By identifying and characterizing of a population-specific genetic determinant of HFpEF, this study may contribute to the understanding of population-specific genetic variants in determining HFpEF phenotypes, particularly in Asian populations.

## Materials and methods

### Ethics declaration

This study adhered to the principles of the Declaration of Helsinki and received ethical approval from the Ethical Committee of the Universitas Airlangga Hospital, Surabaya, Indonesia (Ethical Clearance Number: 159/KEP/2023). All participants agreed to participate and provided written informed consent.

### Study design and population

The sample size and statistical power were estimated using the two-independent-means formula, n = 2(z_1_ ₋ α/_2_ + z_1_ ₋ β)^2^/d^2^, which is commonly used in biomedical research [14,15]. The assumed effect size was derived from a previous study, which reported an approximately 50% reduction in *BAG3* expression in failing human myocardium compared with non-failing human myocardium [16]. Sample size calculations suggested that a minimum total sample size of 50 participants would be required to achieve 80% statistical power, at a significance level of α = 0.05 under the most conservative effect size assumption (*d* = 0.8).

We studied a total of 66 participants (HFpEF: n = 36; control (non-HFpEF individuals): n = 30) of Indonesian origin. Using the same approach as above, the estimated statistical power is approximately ~89%. HFpEF was determined using the diagnostic criteria as outlined in the 2022 AHA/ACC/HFSA and the 2021 ESC Guidelines for the Management of Heart Failure as follows: i) the presence of symptoms and signs of heart failure; ii) normal left ventricular ejection fraction (LVEF ≥ 50%); and iii) evidence of elevated left ventricular filling pressures, either spontaneous or induced, as demonstrated by increased natriuretic peptide levels and/or through non-invasive or invasive hemodynamic assessments [2,17]. Participants were recruited from the Cardiology and Vascular Medicine outpatient clinics of Universitas Airlangga Hospital, Surabaya, Indonesia, between 1^st^ Nov 2023–28^th^ Feb 2024.

### Inclusion and exclusion criteria

The inclusion criteria comprised adult patients with a clinical diagnosis of HFpEF, who were fully conscious and provided informed consent for participation. The exclusion criteria included patients with significant systemic illnesses, uncontrolled cardiac arrhythmias, recent acute coronary syndrome or acute HF, or a history of malignancy or autoimmune diseases. The control group included healthy adults who had no symptoms of HF, no evidence of elevated left ventricular filling pressures, either spontaneous or provoked, as indicated by normal natriuretic peptide levels, and were confirmed to have no history of cardiovascular disease through comprehensive physical examinations and echocardiographic evaluations.

### Clinical and demographic data collection

Demographic and clinical data were gathered through structured interviews and thorough reviews of medical records. Data collection included sex, age, cardiovascular symptoms, and medical comorbidities such as DM, hypertension, and smoking history. Anthropometric measurements were conducted to determine body mass index (BMI), and blood pressure was recorded using a calibrated digital sphygmomanometer. Hypertension was classified following the 2024 ESC Guidelines and 2025 AHA/ACC/AANP/AAPA/ABC/ACCP/ACPM/AGS/AMA/ASPC/NMA/PCNA/SGIM Guidelines [18,19]. Laboratory analyses included serum lipid profiles, specifically low-density lipoprotein (LDL), high-density lipoprotein (HDL), and total cholesterol levels, as well as N-terminal pro B-type natriuretic peptide (NT-proBNP) levels. Echocardiographic evaluations were performed to assess structural and hemodynamic parameters, including left ventricular ejection fraction (LVEF), relative wall thickness (RWT), left ventricular mass index (LVMi), septal mitral annulus velocity (septal e’), lateral mitral annulus velocity (lateral e’), average ratio of early diastolic mitral inflow velocity (E) to early diastolic mitral annulus velocity (e’) or E/e’ ratio, the ratio of early diastolic flow velocity to late diastolic transmitral flow velocity (E/A ratio), and mitral valve E velocity (MV E Vel). The functional capacity of patients with HFpEF was classified using the New York Heart Association (NYHA) functional classification system.

### Sample collection and processing

Blood samples were collected from each participant under standardized conditions using vacutainer tubes. Samples were promptly centrifuged, aliquoted, and stored at -80^o^C until further analysis. Approximately 5 mL of venous blood was drawn from each participant, and peripheral blood mononuclear cells (PBMCs) were extracted from each sample. The plasma was then transferred into a tube for further processing. The DNA was subsequently extracted using the QIAamp DNA extraction kit (Qiagen, Inc., Hilden, Germany), while the RNA was also isolated from the blood using the QIAamp RNA Blood Mini Kit, both according to the manufacturer’s protocols. Next, the obtained RNA was then converted into first-strand cDNA with the High-Capacity cDNA Reverse Transcription Kit (Applied Biosystems).

### Detection of *BAG3* gene polymorphisms

The extracted and isolated DNA was amplified using polymerase chain reaction (PCR) by using Promega GoTaq Green Master Mix. The primers employed in this process were specifically designed to target the region surrounding the rs2234962 polymorphism of the *BAG3* gene (Fig 1). The forward primer sequence was 5’-TTGGCCAAATAGCAACCTCT-3’, and the reverse primer sequence was 5’-AGATGCCCTGCATGTGAA-3’. The expected size of the PCR product was 630 base pairs (bp).

**Fig 1:**
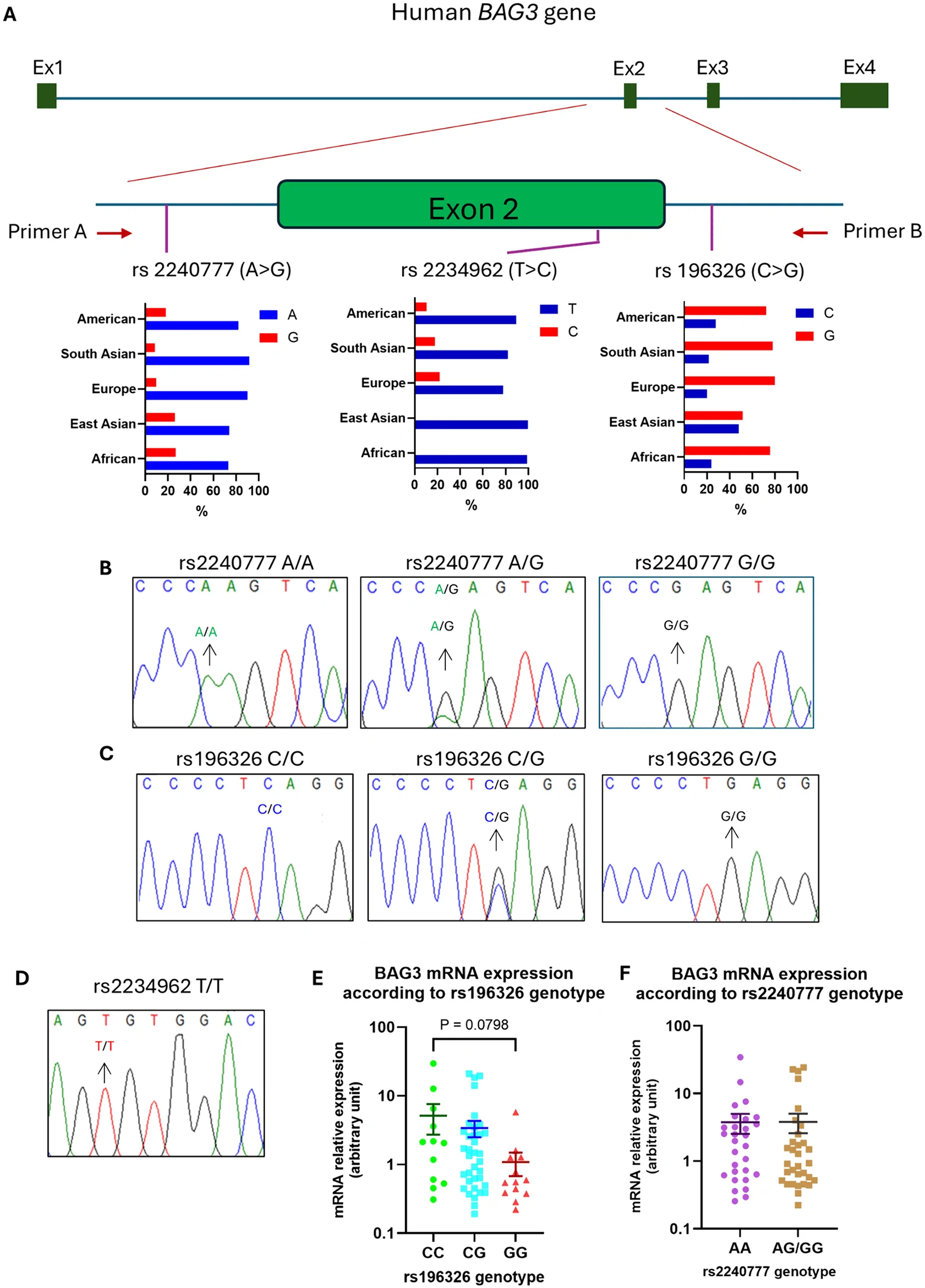
Identification of BAG3 gene polymorphisms. **A)** Diagram depicting the genomic locations of polymorphisms: rs2240777, rs2234962 and rs196326. Known allele frequencies of each SNP according to Genome1000 [12] are shown in the adjacent bar graphs. Arrows indicate the primers used for sequence analysis. **B)** Representative electropherograms indicating the presence of rs2240777 and **C)** rs196326 polymorphisms in our cohort. **D**) However, we only detected the T allele (reference allele) of rs2234962 SNP in our cohort. **E)** Analysis of BAG3 mRNA expressions according to rs196326 SNP (CC,n = 12; CG,n = 36; GG, n = 13) and **F)** based on rs2240777 polymorphism (AA, n = 29; AG/GG, n = 32). Data are presented as mean ± SEM. Statistical test used: E) One way ANOVA followed by multiple comparisons, F) Student’s t-test.

Detection of the BAG3 gene polymorphism was conducted using the Sanger DNA sequencing method. Sequencing results were analyzed using MEGA software (MEGA 2023, Auckland, New Zealand) by comparing the obtained sequences with the reference genomic sequence retrieved from the NCBI Gene database.

### *BAG3* mRNA expression

*BAG3* mRNA expression was quantified using qRT-PCR. The primer sequence used was forward (5′-CGACCAGGCTACATTCCCAT-3′) and reverse (5′-TCTGGCTGAGTGGTTTCTGG-3′). β-actin expression was used as reference. Primers for β-actin qPCR were forward (5’-CAATGAGCGGTTCCGGTGT-3’) and reverse (5’-TGGAGTTGAAGGTGGTCTCG-3’). We used the PowerUp^TM^ SYBR^TM^ Green Master Mix (Applied Biosystem) for qPCR reactions. The expression level of the *BAG3* gene was quantified using the (2^−ΔΔCt^) method and reported as a relative ratio.

### Beclin-1, GRP78, and Caspase-3 plasma levels

The plasma concentrations of Beclin-1, GRP78, and Caspase-3 were measured using validated sandwich enzyme-linked immunosorbent assay (ELISA) kits from Elabscience (Houston, Texas, USA) following protocols recommended by the manufacturer.

### Statistical analysis

Statistical analyses were performed using SPSS version 29 (IBM SPSS, New York, USA) and GraphPad Prism version 10 (GraphPad Software, LLC). Continuous variables are expressed as means ± standard errors of the mean (SEM), while categorical variables are presented as frequencies and percentages. Data normality was assessed using the Shapiro-Wilk test. Inter-group comparisons of normally distributed continuous variables were conducted using independent t-tests, while non-normally distributed variables were analyzed with the Mann-Whitney U test. For more than two groups (e.g., analysis between three genotypes), one way analysis of variance (ANOVA) followed by post-hoc multiple comparisons test was used for normally distributed variables, and the Kruskall Wallis test or the Mann-Whitney U test was used for non-normally distributed variables. Categorical variables were assessed using chi-square or Fisher’s exact tests as appropriate. Statistical significance was defined as p < 0.05 for all analyses. Hardy-Weinberg equilibrium (HWE) was assessed in controls and the combined group for each polymorphism using the χ^2^ goodness-of-fit test [20].

To account for multiple comparisons, p-values were adjusted using the Benjamini–Hochberg false discovery rate (FDR) procedure, and both nominal and FDR-adjusted values are reported. Effect sizes are reported as odds ratios (ORs) with 95% confidence intervals (CIs). Genotype–phenotype associations were further examined using multivariable regression with a dominant genetic model (G-allele carriers [CG + GG] vs non-carriers [CC]), Linear regression was used for log-transformed BAG3 mRNA and caspase-3, and logistic regression for HFpEF occurrence.

### Data availability statement

Individual-level genetic and clinical data cannot be deposited in a public repository since they contain potentially identifiable information. Moreover, the informed consent approved by the institutional ethics committee did not permit unrestricted public release of individual-level data. Data and relevant meta-data will be made available to academic and other researchers after approval of a data access request that can be obtained by email to the corresponding author(s) or to Department of Physiology and Medical Biochemistry, Faculty of Medicine, Universitas Airlangga (biokimia@fk.unair.ac.id). Data access requests will be considered on a case by case basis, subject to a data-sharing agreement consistent with the study's ethical and regulatory requirements.

## Results

### Demographic characteristics of the participants

The demographic characteristics of the participants are summarized in Table 1. No significant differences were observed between the HFpEF and control groups in terms of age, sex, body mass index (BMI), smoking status, blood pressure, history of DM, and cholesterol levels. All patients with HFpEF received treatment with either angiotensin-converting enzyme (ACE) inhibitors or angiotensin receptor blockers (ARBs), with or without diuretics as required. All participants received treatments for more than 6 months. Patients with HFpEF had a mean age of 59.25 ± 11.86 years and a mean BMI of 26.65 ± 4.53 kg/m^2^, classified as obesity class I according to the WHO Asia-Pacific obesity criteria [21]. All patients with HFpEF had shortness of breath or dyspnea. According to the NYHA classification, 6.45% of patients were classified as NYHA class I, 35.48% as NYHA class II, and 58.06% as NYHA class III. No patient had NYHA class IV as our research was conducted in the outpatient unit. The average values of left ventricular mass index (LVMi), relative wall thickness (RWT), and the ratio of early diastolic flow velocity divided by late diastolic transmitral flow velocity (E/A ratio) were higher in the HFpEF group compared to those in the control group, although these differences did not reach statistical significance (Table 2).

**Table 1:** Demographic characteristics of the participants.

Variables	HFpEF n = 36	Control n = 30	Total n = 66	p-Value	FDR
Sex, Female	26 (72.2%)	21 (70%)	47 (71.2%)	0.843^c^	0.943
Age (years)	59.25 ± 11.86	60.93 ± 8.55	60.02 ± 10.45	0.943^b^	0.943
BMI (Kg/m^2^)	26.65 ± 4.53	25.57 ± 4.3	26.16 ± 4.43	0.326^a^	0.890
Smoking					
Smoker or past smoker	6 (16.7%)	7 (23.3%)	13 (19.7%)	0.499^c^	0.890
Non Smoker	30 (83.3%)	23 (76.7%)	53 (80.3%)		
Blood Pressure (mmHg)					
Systolic	133.05 ± 21.57	128.70 ± 18	131.08 ± 20	0.377^a^	0.890
Diastolic	77.47 ± 12.86	79 ± 11.93	78.2 ± 12.38	0.600^a^	0.890
DM History	14 (38.89%)	11 (36.67%)	25 (37.88%)	0.853^c^	0.943
Cholesterol levels (mg/dl)					
HDL-cholesterol	44.83 ± 10.86	49 ± 9.51	46.75 ± 10.41	0.106^a^	0.890
LDL-cholesterol	111.72 ± 40.15	116.3 ± 35.4	113.8 ± 37.85	0.623^a^	0.890
Total cholesterol	173.41 ± 43.91	179.33 ± 33.87	176.1 ± 39.48	0.543^a^	0.890
Treatment					
ACE-inhibitors	3 (8.33%)	3 (10%)			
ARBs	24 (66.67%)	20 (66.67%)			
Diuretics	3 (8.33%)	0 (0)			
Calcium channel blockers	22 (61.11%)	17 (56.67%)			
Insulin	4 (11.11%)	2 (6.67%)			
Oral antidiabetics	8 (22.22%)	6 (20%)			
Statins	16 (44.44%)	12 (40%)			
Beta blockers	28 (77.78%)	0 (0)			

a: independent t-test; b: Mann–Whitney test; c: chi-square test. *FDR-adjusted p-values were calculated across the baseline comparisons using the Benjamini–Hochberg false discovery rate procedure. No variable differed significantly between groups before or after correction.*

**Table 2:** Echocardiography indices of the HFpEF and control groups.

Variables	HFpEF n = 36	Control n = 30	p-Value	FDR
LVEF (%)	62.03 ± 5.15	63.10 ± 5.31	0.410^a^	0.718
LV RWT	0.51 ± 0.16	0.48 ± 0.13	0.360^b^	0.718
E/A ratio	0.95 ± 0.46	0.84 ± 0.22	0.959^b^	0.959
LVMi (g/m^2^)	110.23 ± 33.35	102.42 ± 47.38	0.207^b^	0.718
E/e’	7.55 ± 2.32	7.11 ± 2.02	0.584^b^	0.818
e’ (septal)	0.07 ± 0.02	0.08 ± 0.02	0.135^b^	0.718
MV E Vel	0.61 ± 0.15	0.60 ± 0.16	0.817^b^	0.953

LVEF, left ventricular ejection fraction; LV RWT, left ventricular relative wall thickness; LAVI, left atrium volume index; E/A ratio, ratio of early diastolic flow velocity divided by late diastolic transmitral flow velocity; LVMi, left ventricular mass index; E/e’, ratio of early diastolic mitral inflow velocity to early diastolic mitral annulus velocity, e’ septal, septal mitral annulus velocity; MV E Vel, mitral valve E velocity. ^a^: independent t-test; ^b^: Mann–Whitney test.

### Identification of *BAG3* genetic variations

Previous studies have indicated strong association between *BAG3* single nucleotide polymorphism (SNP) rs223492 and the occurrence of heart failure [7]. This SNP is located in the coding region (exon 2) causing amino acid substitution (C151R). To analyse the presence of this genetic variant in our HFpEF cohort, we performed DNA sequencing analysis of the PCR fragments spanning the exon 2 of *BAG3* gene (Fig 1A). We did not detect the rs2234962 genetic variation in all HFpEF patients (n = 36) and normal individuals (n = 30) tested. However, we detected other SNPs: rs196326 and rs2240777, in both HFpEF and control groups. The rs196326 SNP is a C > G base substitution located at 72 bp downstream of the exon 2, whereas the rs2240777 SNP is an A > G base substitution at 107 bp upstream of the exon 2. The genomic locations of these SNPs are shown in Fig 1A, and the examples of DNA sequencing electropherograms showing the presence of the variants are shown in Fig 1B-D.

The Hardy-Weinberg equilibrium (HWE) was evaluated using the χ^2^ goodness-of-fit test. Both rs196326 and rs2240777 SNPs were in HWE in the control group (P = 0.72 and P = 0.90, respectively) and in the combined study population (P = 0.09 and P = 0.38, respectively), indicating no significant deviation from expected genotype frequencies.

The frequencies of the rs196326 and rs2240777 SNPs in our cohort are shown in Table 3. The frequency of the C allele of the rs196326 SNP in the entire cohort was 0.5, the rs2240777 G allele was 0.289, while no rs223492 polymorphic allele was detected in our cohort. These findings are in line with data from the 1000 Genomes Project and gnomAD [12,13]. The frequency of the rs2234962 polymorphic allele is indeed very low in East Asian populations (0.002), compared with European (0.219), South Asian (0.179), American (0.104) and African (0.011) populations. The frequency of the rs196326 polymorphism was higher in East Asian populations, with the C allele frequency of 0.483, compared to other populations, such as European (0.200), African (0.242), American (0.277) and South Asian (0.218). Similarly, the frequency of the rs2240777 polymorphism (G allele) was more common in East Asians (0.261), compared to European (0.098), American (0.180) and South Asian (0.086)(Fig 1A).

**Table 3:** Frequency of *BAG3* gene polymorphisms in the HFpEF and control groups.

*BAG3* polymorphism		HFpEF (n = 36)	Control (n = 30)	p-value
**rs196326**				
**Genotype**	**C/C**	6 (16.67%)	7 (23.33%)	0.544
	**C/G**	24 (66.67%)	16 (53.33%)	
	**G/G**	6 (16.67%)	7 (23.33%)	
**Dominant genetic model**	**C/C**	6 (16.67%)	7 (23.33%)	0.498
	**C/G + G/G**	30 (83.33%)	23 (76.66%)	
**rs2240777**				0.793
**Genotype**	**A/A**	16 (44.44%)	16 (53.33%)	
	**A/G**	18 (50%)	12 (40%)	
	**G/G**	2 (5.56%)	2 (6.67%)	
**Dominant genetic model**	**A/A**	16 (44.24%)	16 (53.33%)	0.472
	**A/G + G/G**	20 (55.76)	14 (46.67%)	

### rs196326 polymorphism is associated with *BAG3* expression

We performed qRT-PCR analysis on total RNA isolated from the peripheral blood mononuclear cells (PBMCs) to assess the level of *BAG3* mRNA expression in both the HFpEF and control groups. We then examined the association between rs196326 and rs2240777 polymorphisms and the *BAG3* mRNA level in all study participants. We found that there was a strong trend of *BAG3* reduction with the presence of the G allele of the rs196326 SNP, with individuals bearing the GG genotype showing a trend of reduced *BAG3* expression compared to those with the CC genotype (P = 0.07)(Fig 1E), indicating the functionality of this SNP in determining *BAG3* expression. In contrast, there was no difference in *BAG3* expression between individuals with AA genotype of the rs2240777 SNP compared to those with the AG/GG genotypes (Fig 1F).

### The rs196326 and rs2240777 SNPs did not correlate with the occurrence of HFpEF

Next, we analysed whether the two SNPs, that were identified in Indonesian patients, were associated with the occurrence of HFpEF. In contrast to the rs2234962 SNP, which has been found to be strongly correlated with the incidence of HF [7], we did not find any correlation between rs196326 and rs2240777 polymorphisms and the occurrence of HFpEF (Table 3).

Consistently, effect sizes for disease occurrence were small and their confidence intervals included the null value (unadjusted: rs196326 G-carrier OR: 1.52, 95%, CI 0.45–5.15; rs196326 allelic OR: 1.00, 95%, CI 0.50–1.98; rs2240777 carrier OR: 1.43, 95% CI 0.54–3.78; rs2240777 allelic OR: 1.21, 95%, CI 0.57–2.59). In multivariable logistic regression adjusting for age, sex, and DM, neither SNP was associated with HFpEF occurrence (rs196326 G-carrier adjusted OR: 1.48, 95% CI 0.43–5.14, P = 0.53; rs2240777 G-carrier adjusted OR: 1.62, 95% CI 0.58–4.53, P = 0.36), and no comparison remained significant after FDR adjustment

### Association of *BAG3* rs196326 Polymorphism with echocardiographic indices

The possible involvement of the rs196326 SNP in determining BAG3 expression level prompted us to hypothesize that this variant may be associated with HFpEF phenotypes. Therefore, we analysed whether there was any correlation between the rs196326 SNP and the cardiovascular indices of patients with HFpEF. Although there was no difference in LVMi between the rs196326 genotype groups (Fig 2A), we found a reduction in the RWT of patients with the CG and GG genotypes (Fig 2B). There was no difference in ejection fraction (EF), mitral valve E velocity (MV E Vel) and systolic blood pressure; however, the E/A ratio was significantly lower in the CG and GG groups (Fig 2C-F). Overall, the data suggested that the rs196326 polymorphism, which affected BAG3 mRNA expression, was associated with some echocardiography parameters and hence the cardiac phenotypes of HFpEF.

**Fig 2:**
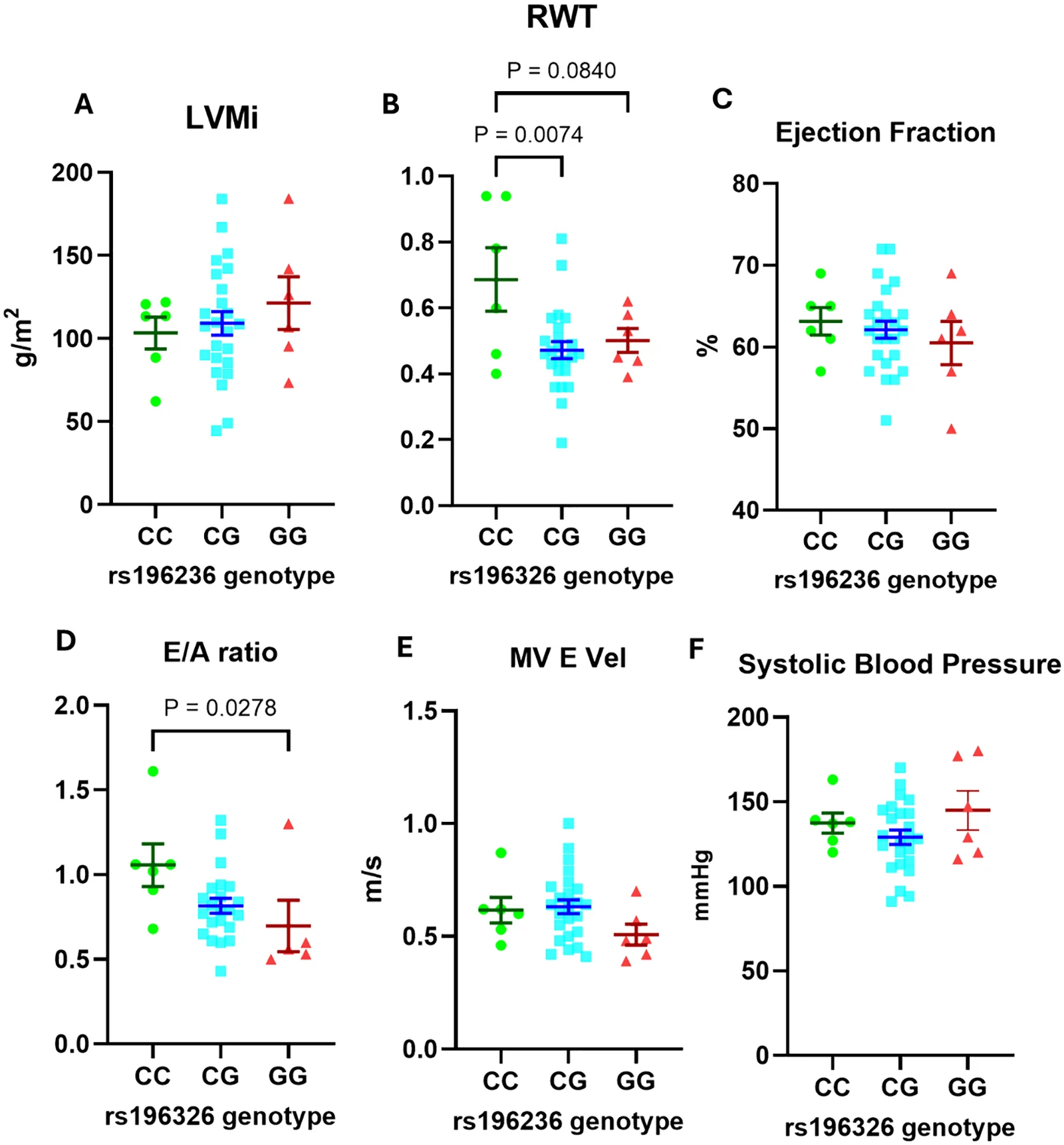
Echocardiography indices of patients with HFpEF according to rs196326 polymorphism. Echocardiography analyses were performed in HFpEF patients to assess **A)** Left ventricular mass index (LVMi), **B)** Relative wall thickness (RWT), **C)** Ejection fraction (EF), **D)** E/A ratio, and **E)** Mitral valve early velocity (MV E Vel), comparing individuals with the CC (n = 6), CG (n = 24) and GG (n = 6) genotype of the rs196326 SNP. **F)** Systolic blood pressure was also examined comparing patients with rs196326 genotypes. Data are presented as mean ± SEM. Statistical tests used: A,B,C,E,F, One-way ANOVA followed by multiple comparisons test; D, Kruskal-Wallis test followed by multiple comparisons.

Multivariable linear regression analysis adjusted for clinical covariates (sex, age, BMI, DM history, smoking status, blood pressure, and lipid profile) yielded results consistent with the unadjusted data shown in Fig 2B. Participants carrying the rs196326 CG genotype demonstrated significantly lower RWT values compared to individuals with the CC genotype (adjusted B=−0.124; 95% CI: −0.244 to −0.004; P = 0.042), while other variables did not show any significant correlations (Table 4). In addition, the multivariate analysis comparing participants with CC vs CG/GG genotypes indicated a strong trend association between the rs196326 polymorphism and RWT (adjusted B=−0.091; 95% CI: −0.193 to 0.011; P = 0.08)(Table 5). These findings suggest that the association of the rs196326 SNP with RWT is independent of potential confounding factors.

**Table 4:** Multivariate regression analysis of RWT and E/A ratio of individuals with rs196326 CC vs CG genotypes.

Variables	RWT			E/A Ratio		
	B	95% CI	P-value	B	95% CI	P-value
Sex	0.007	−0.133 - 0.146	0.924	−0.134	−0.449 - 0.182	0.398
Age	−0.001	−0.006 - 0.004	0.712	−0.017	−0.028 - −0.006	0.003
BMI	0.008	−0.003 - 0.020	0.158	−0.006	−0.032 - 0.020	0.659
Type 2 Diabetes	0.050	−0.053 - 0.152	0.333	−0.053	−0.284 - 0.179	0.649
Systolic BP	0.002	−0.002 - 0.005	0.280	−0.002	−0.010 - 0.007	0.706
Diastolic BP	−0.002	−0.007 - 0.004	0.520	−0.003	−0.015 - 0.010	0.665
Smoking	−0.097	−0.254 - 0.060	0.219	0.028	−0.326 - 0.382	0.875
HDL Cholesterol	−0.001	−0.007 - 0.004	0.578	0.004	−0.007 - 0.016	0.439
LDL Cholesterol	0.000	−0.002 - 0.002	0.742	0.002	−0.003 - 0.007	0.425
Total Cholesterol	0.001	−0.001 - 0.004	0.199	−0.001	−0.006 - 0.004	0.615
rs196326	−0.124	−0.244 - −0.004	0.042	0.071	−0.199 - 0.342	0.597

**Table 5:** Multivariate regression analysis of RWT and E/A ratio of individuals with rs196326 CC vs CG/GG genotypes.

Variables	RWT			E/A Ratio		
	B	95% CI	P-value	B	95% CI	P-value
Sex	0.025	−0.096–0.146	0.678	−0.140	−0.419–0.1390	0.318
Age	−0.002	−0.006–0.002	0.231	−0.016	−0.025 – −0.007	0.001
BMI	0.006	−0.002–0.015	0.151	0.004	−0.016–0.024	0.715
Type 2 Diabetes	0.011	−0.068–0.090	0.779	−0.059	−0.241–0.124	0.521
Systolic BP	0.002	−0.001–0.005	0.263	0.000	−0.007–0.006	0.942
Diastolic BP	−0.001	−0.006–0.004	0.578	−0.002	−0.014–0.009	0.713
Smoking	−0.075	−0.210–0.060	0.272	0.040	−0.272–0.351	0.800
HDL Cholesterol	−0.001	−0.005–0.003	0.589	0.009	−0.001–0.019	0.082
LDL Cholesterol	0.000	−0.001–0.002	0.698	0.002	−0.001–0.006	0.211
Total Cholesterol	0.000	−0.001–0.002	0.605	−0.001	−0.005–0.003	0.543
rs196326	−0.091	−0.193–0.011	0.080	0.072	−0.164–0.309	0.541

In contrast, multivariable linear regression analysis for the E/A ratio demonstrated that the association between the rs196326 polymorphism and the E/A ratio was not independent of potential confounders. No significant associations were observed when comparing CC vs. CG groups (adjusted B = 0.071; 95% CI: −0.199 to 0.342; P = 0.597) or CC vs. CG/GG genotypes (adjusted B = 0.072; 95% CI: −0.164 to 0.309; P = 0.541)(Tables 4 and 5). Notably, the significant difference in the E/A ratio between CC and GG observed in the unadjusted comparison (Fig 2D, P = 0.0278) was attenuated after adjusting for confounders, particularly age (adjusted B=−0.023; P = 0.038). These findings indicate that the unadjusted association with the E/A ratio was largely driven by age-related confounding rather than a direct genetic effect of rs196326.

### *BAG3* rs196326 SNP alters the level of some serum biomarkers

Since *BAG3* has been identified as a modulator of several important cellular processes such as ventricular remodelling, autophagy, and apoptosis [22], we next assessed whether the *BAG3* rs196326 polymorphism is associated with some key biomarkers such as NT-proBNP (marker of HF and ventricular distress)(Fig 3A), Beclin1 (autophagy marker)(Fig 3B), caspase 3 (apoptosis/metabolic stress)(Fig 3C), and glucose regulated protein 78 (GRP78, marker of endoplasmic reticulum stress)(Fig 3D) levels. The CG and GG genotypes, which were associated with lower *BAG3* expression, were correlated with higher NT-proBNP levels, indicating more severe ventricular wall stress. However, the rs196326 genotypes were not associated with the autophagy marker Beclin-1 and endoplasmic reticulum (ER) stress marker GRP78. Interestingly, individuals with CG/GG genotypes displayed lower caspase-3 levels, suggesting a reduction in apoptosis or metabolic stress.

**Fig 3:**
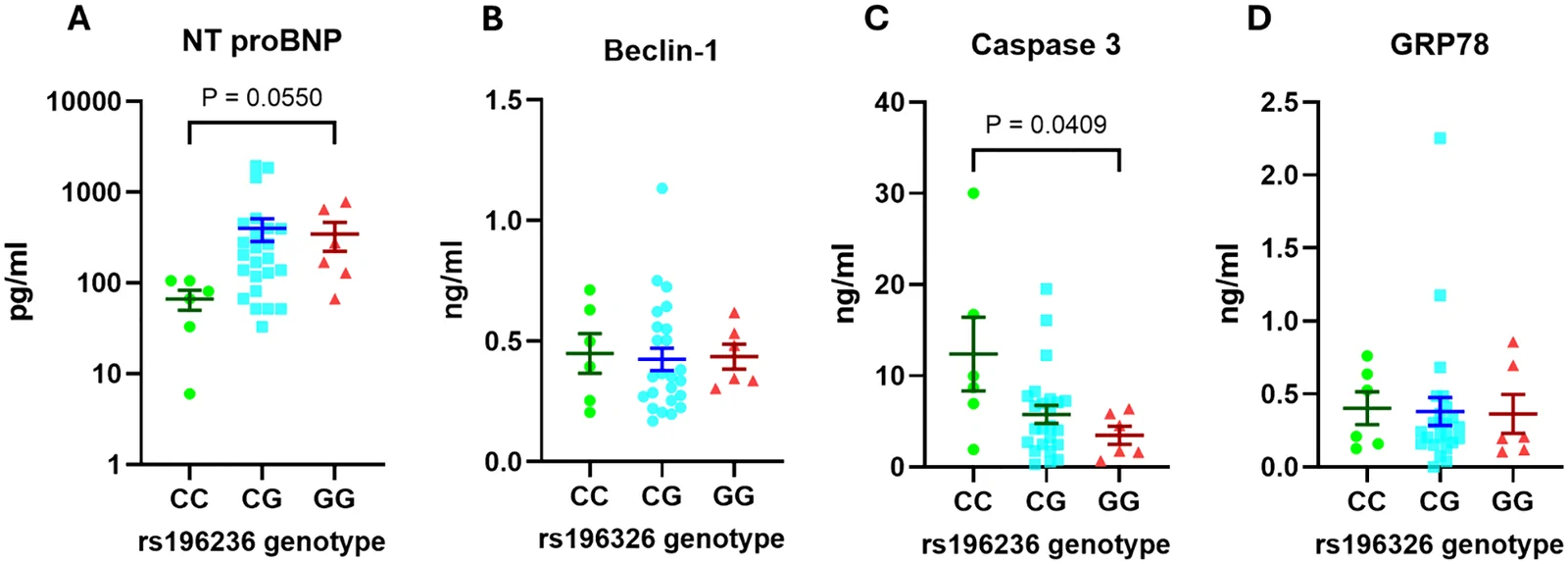
Analysis of serum biomarkers in HFpEF patients according to rs196326 genotype. Serum biomarkers were examined using ELISA in patients with HFpEF, including **A)** Heart failure marker NT-proBNP, B) Autophagy biomarker Beclin-1, C) Caspase-3 (marker of apoptosis and metabolic stress), and D) GRP78 (biomarker of ER stress). Comparisons were performed according to rs196326 genotypes (CC,n = 6; CG,n = 24; GG,n = 6). Data are presented as mean ± SEM. Statistical test used: A,B,C,D, Kruskal-Wallis test followed by multiple comparisons.

### *BAG3* rs2240777 did not correlate with echocardiographic parameters

Contrary to the rs196326 SNP, the rs2240777 polymorphism showed no correlation with the cardiac phenotypes of patients with HFpEF, including the LVMi, RWT, EF, MV E Vel and E/A ratio (Fig 4A-F). This was in line with the finding that rs2240777 did not correlate with *BAG3* mRNA levels. However, we found that the AG/GG genotypes of the rs2240777 polymorphism were associated with higher serum NTproBNP levels, but lower caspase-3 levels compared to individuals with the AA genotype. However, no differences were observed in Beclin1 or GRP78 biomarker levels for rs2240777 (Fig 5A-D).

**Fig 4:**
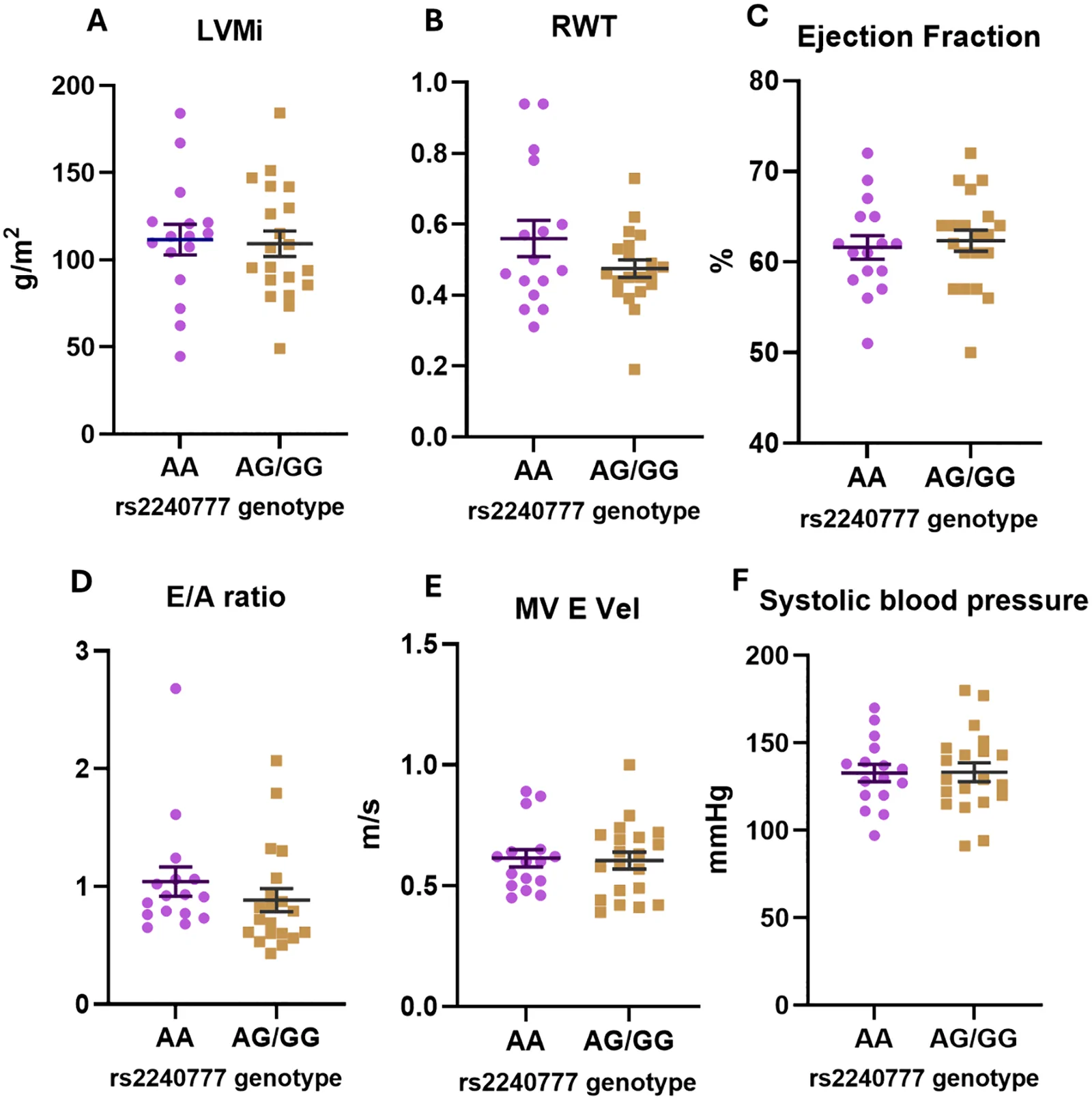
Echocardiography analyses comparing HFpEF patients with different rs2240777 genotypes. Analysis of cardiac morphology and functions was performed to compare several echocardiography parameters including the **A)** Left ventricular mass index (LVMi), **B)** Relative wall thickness (RWT), **C)** Ejection fraction (EF), **D)** E/A ratio, and **E)** Mitral valve early velocity (MV E Vel), between patients with rs2240777 genotypes: AA (n = 16) and AG/GG (n = 20). **F)** Systolic blood pressure was also examined comparing patients with the rs2240777 AA vs AG/GG genotypes. Data are presented as mean ± SEM. Statistical tests used: A,C,F, Student’s t-test; B,D,E, Mann-Whitney U test.

**Fig 5:**
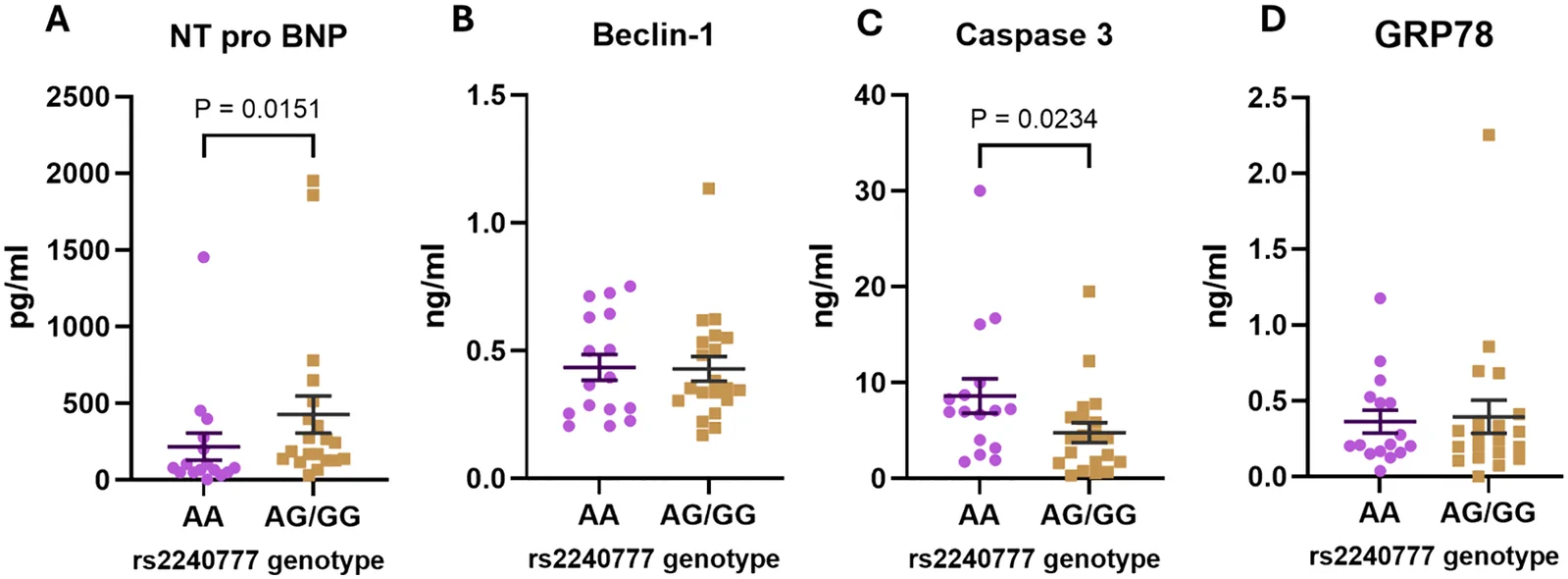
Levels of clinical and pathophysiological biomarkers according to rs2240777 genotypes. Biomarkers of **A)** Heart failure and myocardial stretch (NT-proBNP), B) Autophagy (Beclin-1), C) Apoptosis and metabolic stress (caspase-3), and D) Endoplasmic reticulum (ER) stress (GRP78) were analysed and compared between HFpEF patients with the rs2240777 AA genotype (n = 16) and AG or GG genotypes (n = 20). Data are presented as mean ± SEM. Statistical tests used: A,B,C,D, Mann-Whitney U test.

### Bioinformatics analyses to understand possible molecular mechanisms

We used bioinformatic tools to understand the possible mechanism(s) by which the rs196326 SNP affects *BAG3* expression. Since this SNP is located near the exon-intron boundary, we studied data from SpliceAI that could predict changes in mRNA splice sites due to the SNP [23]. Fig 6A shows the predicted splice sites (splice donors and acceptors) adjacent to rs196326. However, the SpliceAI algorithm did not predict any significant changes between the reference and alternate scores, indicating that the SNP is not likely to alter splice sites, either by disrupting or by creating splice sites.

**Fig 6:**
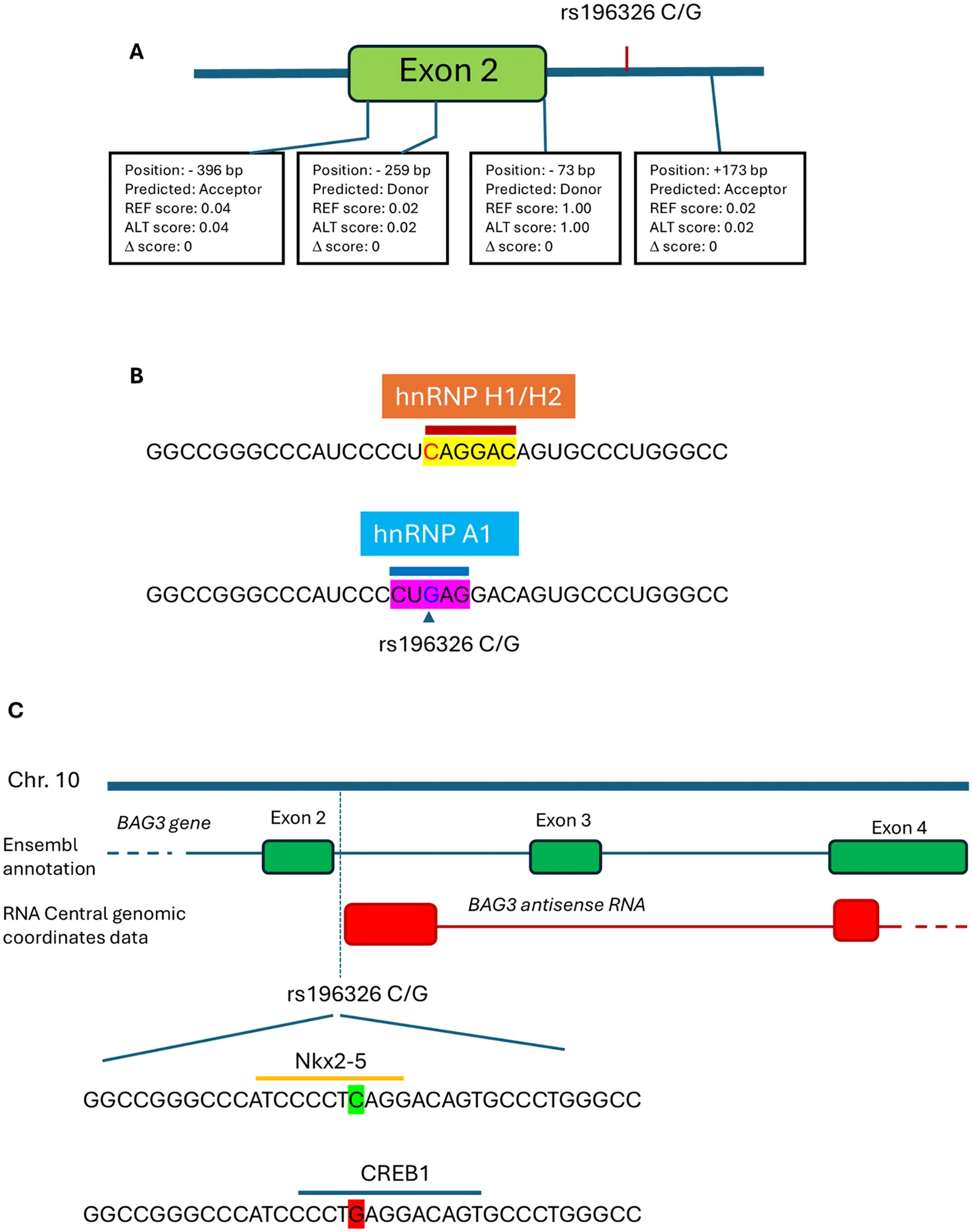
Computational analysis to predict the molecular processes affected by the rs196326 SNP. **A)** Data from SpliceAI describing that the rs196326 genetic variation does not significantly alter splicing donors or acceptors near the polymorphic site. B) SpliceAid2 algorithm was used to predict the binding of intronic splicing enhancers (ISE) and intronic splicing silencers (ISS) at the polymorphic site. The data indicate that rs196326 C/G variations may change the likelihood of binding to different ISE/ISS, from binding with hnRNP H1/H2 (C allele) to hnRNP A1 (G allele). C) The rs196326 SNP may alter the binding to transcription factors/enhancers. By using Ciiider it was predicted that the reference sequence carrying C allele favours binding to Nkx2-5, whereas the alternate G allele favours binding to CREB. The RNA genomic data shows that this sequence is adjacent to the transcribed *BAG3* antisense RNA region suggesting a possible alteration in BAG3 gene expression regulation.

The intronic sequence near splice sites may be involved in RNA splicing regulation by binding to intronic splicing enhancers (ISE) or intronic splicing silencers (ISS). We therefore used the SpliceAid2 database [24] to analyse if nucleotide substitution in rs196326 site alters ISE or ISS binding. As shown in Fig 6B, SpliceAid2 predicted significant changes in ISE/ISS binding patterns, as the sequence bearing the C allele likely binds to splicing regulators hnRNP H1/H2, whereas the polymorphic allele was more likely to bind to a splicing silencer (hnRNP A1).

Intronic sequence variations may also affect binding to transcription factors (TFs) or enhancers (TEs). Data from RNAcentral [25] suggest that a long non-coding RNA that can functions as antisense mRNA against *BAG3* is located within the human *BAG3* gene, adjacent to the rs196326 SNP site (Fig 6C). We then analyse if rs196326 SNP alters the binding of TFs or TEs on this site. By using Ciiider [26], a tool that can predict TF binding sites, we found that there is a possible alteration of TFs binding favouring Nkx2–5 in the reference allele to more likely binding to CREB in the polymorphic allele. This eventually may affect the expression of the antisense RNA that regulates *BAG3* expression. However, it is important to note that these predictions are entirely based on in silico modelling and have not been experimentally validated. Direct functional experiments (e.g., luciferase reporter assays, minigene splicing assays, or CRISPR-based genome editing) are required to establish a regulatory mechanism.

## Discussions

The main finding of this study is that the rs196326 polymorphism within the human *BAG3* gene may potentially determine cardiac phenotypes among HFpEF patients of Indonesian ethnicity. In this study we detected two intronic variants of human *BAG3* gene, the rs196326 and rs2240777, as the predominant genetic polymorphisms in this population. In contrast, the widely studied *BAG3* polymorphism (rs2234962), which is frequently reported in Caucasian cohorts and is associated with HF and cardiomyopathy [7,9,10], was not detected in our cohort. This observation is consistent with data from available polymorphisms databases showing the extremely low frequency of the minor allele of these polymorphisms in East Asian populations.

Unlike the rs2234962 SNP, we did not observe any association between the rs196326 and rs2240777 SNPs and HFpEF occurrence. This may be due to the nature of the SNPs, in which the rs2234962 alters a protein coding region, whereas both the rs196326 and rs2240777 are located in non-coding regions, which may reduce any functional effect. However, it is important to note that the current study was only conducted among a small number of participants; hence, the statistical power might be limited. A much larger case-control study is required to confirm the association of these SNP with the occurrence of HFpEF,.

In our cohort, the rs196326 polymorphism demonstrated a clear genotype-phenotype associations with the cardiac phenotypes of HFpEF patients. Our observations suggest that individuals with the polymorphic allele have reduced relative ventricular wall thickness (RWT) despite a preserved LV mass index (LVMi), indicating a possible eccentric hypertrophy phenotype. A lower RWT could be due to the thinning of the LV wall and/or increased chamber dimension (dilatation), and it is often associated with a more severe HF phenotype. This is in line with the finding that individuals carrying the rs196326 polymorphic allele (the G allele) displayed higher NT-proBNP levels, a marker of cardiac wall stretch [27]. The lower RWT observed in patients with the polymorphic allele implies a mechano-adaptive signaling response, leading to a ventricular geometry that is less capable of countering increased filling pressures, despite a preserved ejection fraction. Since RWT depends on both wall thickness and chamber dimension, a mild reduction in the RWT without significant chamber dilatation could leave the LVMi unchanged [28]. This pattern is contrary to patients with the CC genotype who mostly exhibited a higher RWT (concentric hypertrophy adaptation). This may indicate that these individuals might have a higher capacity for compensatory mechanism against chronic pressure overload.

In keeping with the morphological indices, we also observed that patients with the G allele displayed a lower E/A ratio, suggesting a more severe diastolic dysfunction. A lower E/A ratio could be interpreted as impaired relaxation and/or increasde atrial contribution during diastole to compensate for the reduction in passive ventricular relaxation [29]. Together, our findings imply that the presence of the rs196326 G allele is associated with a worse cardiac phenotype.

Importantly, we found that participants carrying the G allele of the rs196326 variant exhibited a trend of lower *BAG3* mRNA expression. As an adaptor protein and co-chaperone, *BAG3* has multiple functions. It is involved in mediating autophagy through its chaperone activity in identifying and transferring damaged proteins/organelles to autophagosomes [22]. In skeletal muscle cells and cardiomyocytes, *BAG3* is essential in preserving the structural integrity of the sarcomeres and stabilizing the cytoskeleton, particularly through its interactions with chaperones such as HSPB8, HSP70, and αB-crystallin localized at the Z-disc [30]. A previous study showed that lower *BAG3* levels decreased contractile force generation and increased myofilament ubiquitination despite preserved sarcomere morphology, while *BAG3* gene therapy in a mouse model of post-infarction heart failure fully improved the condition [31]. Other cellular processes regulated by *BAG3* include apoptosis, excitation-contraction coupling and intracellular transport [32]. Therefore, it is logical that decreased *BAG3* is associated with detrimental effects, as demonstrated in studies using knockout mice, in which cardiomyocte-specific knockout of this gene led to dilated cardiomyopathy, severe fibrosis and premature death [16].

Interestingly, the serum levels of caspase-3 were lower in patients with the rs196326 CG and GG genotypes, suggesting that this variant may exert anti-apoptotic effects. This is contrary to the finding that *BAG3* plays a role in blocking apoptosis via its interactions with HSP70 and BCL2, enhancing cardiomyocyte survival under stress conditions [33]. Lower serum caspase-3 may indicate that systemic apoptosis process is less active. Caspase-3 is also associated with metabolic stress [34]. However, changes in serum caspase-3 levels do not inform specifically which organ that was affected. There is also a possibility that unexpected low caspase-3 levels may indicate a lack of stress response in severe pathological conditions. For example, patients with severe stroke may have low serum caspase-3 levels, because less caspase-3 is secreted in acute stroke with lack of reperfusion [35].

Although the mechanism is not completely understood, there is a possible paradox situation where caspase-3 is lower in worse phenotypes. As we measured caspase-3 in peripheral blood mononuclear cells (PBMCs), it reflects systemic and apoptotic and inflammatory conditions of circulating cells rather than local myocardial apoptosis. HFpEF is a systemic disorder in which comorbidities such as obesity, diabetes, and hypertension drive a chronic low-grade inflammatory and metabolic state that leads to endothelial inflammation and myocardial remodelling [36]. Interestingly, a study by Bott-Flugel et al showed that caspase-3 can be differently expressed in acute and chronic HF. In this study, an increase in caspase-3 was only observed in patients with acute HF, while it was lower in chronic HF [37]. Our finding of concomitantly lower plasma *BAG3* and lower total serum caspase‑3 in patients with HFpEF is consistent with a phenotype of relatively stable, chronic heart failure rather than an acute condition [38].

We also examined other serum markers such as Beclin1 (autophagy marker) and GRP78 (ER stress marker). We assessed these proteins because *BAG3* is a known modulator of autophagy; hence, it is involved in ER stress regulation. However, we did not observe any difference in these markers, suggesting that in the setting of HFpEF, the difference in *BAG3* levels due to the rs196236 genetic variation may not affect autophagy and ER stress in the heart.

The next question is how the rs196326 SNP affects *BAG3* expression. This polymorphism is located in an intronic region near the 3’ end of the *BAG3* exon 2. There are several possible mechanisms as to how an intronic SNP affects gene expression. It may alter RNA splicing mechanism by: i) disrupting the splice sites (either the splice donors or acceptors); ii) creating new cryptic splice sites; and iii) changing the binding of splicing enhancers (ISE) or splicing silencers (ISS). Using available computational algorithms, we found that the rs196326 SNP more likely alters ISE/ISS binding rather than disrupting or creating splice sites. The sequence carrying the C allele is predicted to bind with hnRNP H1/H2, which are known as major splice site regulators and may act as enhancers or silencers [39]. Conversely, the sequence bearing the G allele is predicted to bind with hnRNP A1, which is known as a major splicing silencer [40].

The other alternative mechanism is through alteration of transcription factor (TF)/enhancer (TE) binding. The rs196326 SNP may alter TFs binding from the likelihood to bind Nkx2–5 (C allele) to more tendency to bind CREB (G alelle). Indeed, the rs196326 SNP is located at the non-coding intronic region of BAG3 gene; however, data from RNAcentral [25] shows that it is situated upstream of a sequence that is transcribed into *BAG3* antisense non-coding RNA. This may affect the expression level of *BAG3*. Despite these possible predicted mechanisms, further experiments are needed to provide direct evidence of the molecular process underlying the functionality of the rs196326 SNP.

In contrast to the rs196326 SNP, the rs2240777 polymorphism showed a different pattern of effects, with no association with *BAG3* mRNA expression or cardiac phenotypes. However, rs2240777 was significantly associated with increased NT-proBNP levels and decreased caspase-3 levels, mirroring the stress and apoptotic signatures observed in rs196326, albeit without detectable changes in ventricular remodeling. Unlike rs196326, the rs2240777 SNP is unlikely to have a significant regulatory effect on *BAG3* transcription in this study population. These findings further underscore the idea that the functional consequences of genetic variation in *BAG3* are variant-specific and may require investigation in larger cohorts or with complementary functional assays to clearly elucidate the molecular mechanism.

Overall, these findings illustrate that *BAG3* variants among Indonesian population may influence HFpEF phenotypes through pathways involving the response to myocardial wall stress. HFpEF is a complex condition caused by multiple factors, and genetic influences often contribute in subtle ways that interact with comorbidities, aging, and environmental stress. In this context, the observation that *BAG3* variants can modify NT-proBNP and caspase-3 levels without causing clear changes in ventricular mass or systolic function is unsurprising and aligns with the gradual, heterogeneous progression typically seen in HFpEF. These findings also raise the possibility that *BAG3* polymorphisms may help distinguish patient subgroups with different capacities to adapt to myocardial stress or differing risks for clinical progression.

From a population genetics perspective, the rs196326 SNP is more frequently observed in Asians than the more commonly studied rs2234962 polymorphism, which is more prevalent in European populations. This disparity likely reflects evolutionary differences as to how the *BAG3* gene modulates stress adaptation across populations. It is plausible that individuals with East or Southeast Asian background carrying the minor allele of rs2234962 polymorphism have a survival disadvantage leading to an extremely low frequency of the minor allele. In contrast, while the frequency of the rs196326 SNP in European, American and African ethnicities is comparable to that of Asians, it is not known as to whether this SNP is also correlated with the severity of diseases such as heart failure. However, the finding that these two SNPs with two different mechanisms of actions (rs2234962 causes amino acid substitution and hence alters BAG3 function while rs196326 reduces *BAG3* expression) are associated with heart failure phenotypes, underscore the importance of *BAG3* gene in cardiac pathological processes. Further studies are needed to understand if BAG3 can become new target of therapy.

A further consideration is that *BAG3* mRNA was quantified in PBMCs rather than in cardiac tissue. PBMCs are an accessible and commonly used surrogate component, and their transcriptional profiles have been shown to carry relevant signatures with diagnostic and prognostic value in HF, according to previous studies [41,42]. However, further studies using relevant tissues or cellular models (e.g., induced pluripotent stem cell derived cardiomyocytes) are needed to confirm the finding.

Our study had several limitations. First, the sample size was relatively small and drawn from a single center; hence, the number of individuals with a particular genotype was small, which may limit the statistical power and generalizability of the findings. However, our power calculation analysis indicated acceptable statistical power despite the small number of participants. Second, the cross-sectional design prevents the drawing of causative interpretations between the *BAG3* variants and the development of HFpEF. Third, although all participants in this study had antihypertensive treatment for more than 6 months, treatment dosing was not systematically captured in this study; therefore, it could not be analysed retrospectively. Fourth, functional assays for the identified SNPs, which may provide direct evidence on the influence of these SNPs on the expression of *BAG3* gene or activity of the protein, were also not performed. However, our findings underscore the relevance of *BAG3* intronic variation as a potential modifier of HFpEF pathobiology and highlight the need for larger, multi-center studies incorporating functional assays to clarify the mechanistic significance and clinical applicability of these population-specific variants. Therefore, our findings should therefore be regarded as hypothesis-generating and require independent validation in larger, multi-centre cohorts incorporating multivariable adjustment and direct functional assays, along with prospective evaluation of detailed medication data.

In conclusion, this study demonstrates that *BAG3* genetic variation in an Indonesian HFpEF population is characterized predominantly by the intronic polymorphisms rs196326 and rs2240777, while the commonly reported coding variant rs2234962 was absent. Among these variants, rs196326 showed a significant association with *BAG3* mRNA expression. Furthermore, the polymorphic allele, which is associated with lower *BAG3* expression, correlates with more severe HFpEF phenotypes, including eccentric remodelling, severe diastolic dysfunction, and elevated NT-proBNP levels. Given the ethnic-specific distribution of these variants, larger multi-center studies with longitudinal follow-up and functional analyses are warranted to validate these observations and clarify their mechanistic and clinical implications.
